# Use of the Tubridge flow diverter in the treatment of intracranial aneurysms: a single center experience

**DOI:** 10.1038/s41598-024-57840-2

**Published:** 2024-03-27

**Authors:** Li Li, Bu-Lang Gao, Qiao-Wei Wu, Qiu-Ji Shao, Zi-Liang Wang, Kun Zhang, Tian-Xiao Li

**Affiliations:** Henan Provincial People’s Hospital, Zhengzhou University, 7 Weiwu Road, Zhengzhou, Henan Province China

**Keywords:** Intracranial aneurysms, Tubridge flow diverter, Endovascular embolization, Complications, Follow-up, Diseases, Health care, Neurology

## Abstract

To investigate the safety and effect of Tubridge flow diverter deployment for the treatment of intracranial aneurysms, 85 patients with intracranial aneurysms treated with the Tubridge flow diverter were retrospectively enrolled. The clinical data including the baseline data, aneurysm parameters before and after treatment, and follow-up outcomes were assessed. Among 85 patients, there were 35 (41.2%) males and 50 females (58.8%) aged 17–77 (mean 56.7 ± 11.1) years with 110 aneurysms. Five (5.9%) patients initially presented with subarachnoid hemorrhage from aneurysm rupture. The aneurysm size was 2–30 (mean 8.6) mm, and the aneurysm neck was 2–10.6 (mean 5.7 ± 2.3) mm. Ninety-three Tubridge stents were deployed. Twenty-five (29.4%) patients experienced adjunctive loose coiling. Blood flow was significantly reduced from entering the aneurysm after stent deployment. Periprocedural complications occurred in three (3.5%) patients, including in-stent thrombosis during embolization in one patient (1.2%), conjunctiva edema on the right in one patient (1.2%), and acute multiple cerebral infarctions in one patient (1.2%). Angiographic follow-up was conducted in 67 (78.8%) patients 3–36 (mean 15.3 ± 5.6) months later. In 11 (16.4% or 11/67) patients, blood flow still entered the aneurysm with the O’Kelly-Marotta (OKM) grade B in two (3.0%) patients and grade C in nine (13.4%), whereas complete occlusion (OKM grade D) was achieved in the other 56 (83.6% or 56/67) aneurysms. In-stent stenosis was present in five (7.5%) patients with approximately 25% stenosis in three (4.5%) patients and 50% in two (3.0%). In conclusion, the Tubridge flow diverter can be safely and efficiently applied in the treatment of small and large intracranial aneurysms, with a low periprocedural complication rate, a high occlusion degree, and a low in-stent stenosis rate at follow-up even though large aneurysms may necessitate a longer surgical time and adjunctive coiling.

## Introduction

Flow diverters have dense woven wire structures and can direct blood flow away and promote thrombosis within cerebral aneurysms after deployment across the aneurysm neck, resulting in subsequent aneurysm occlusion^1,2^. Ever since the Pipeline embolization device (Medtronic, Irvine, CA, USA), the first flow diverter, was approved for clinical use in 2008, an increasing number of cerebral aneurysms have been treated with flow diverters, especially large, giant, and irregular aneurysms^1,3–5^. With experience accumulation, the indications for use of flow diverters have been expanded, and the flow diverter has been increasingly applied to treat very small, small, wide-necked, dissecting, complex, ruptured cerebral aneurysms and aneurysms of small vessels^3–11^, leading to good outcomes. The mechanism of flow diverters in treating cerebral aneurysms involves directing blood flow away from the aneurysm, slowing flow into the aneurysm cavity, promoting thrombosis within the aneurysm, and providing a scaffold at the aneurysm neck for endothelialization to resume the integrity of arterial wall. Moreover, flow diverters may induce parent artery straightening like other common intracranial stents and subsequent alteration of hemodynamic stresses, which can further promote aneurysm thrombosis and occlusion^12–15^. Compared with other endovascular therapeutic approaches, the advantages of simpler operation, higher aneurysm occlusion rates and lower aneurysm recurrence rates of the flow diverters have also promoted their use in the treatment of other more common aneurysms. Very small and small aneurysms need no adjunctive coiling after deployment of a flow diverter. Currently, different flow diverters are available on the market, and the Tubridge stent or vascular reconstruction device (MicroPort, Shanghai, China) is a flow diverter developed in China. Initial clinical studies have shown that this flow diverter is comparable to other ones like the Pipeline flow diverter in the safety and effect for the treatment of large, giant or recurrent intracranial aneurysms^1,16–19^. Nonetheless, experience has not been accumulated sufficiently for the use of the Tubridge stent in the treatment of more common cerebral aneurysms. It was hypothesized that the Tubridge stent could be safely and efficiently applied in the treatment of common aneurysms. This study was consequently performed to explore the safety and effect of the Tubridge stent in treating common cerebral aneurysms.

## Materials and methods

### Subjects

This retrospective single-center study was approved by the ethics committee of Henan Provincial People’s Hospital, and because of the retrospective study design, informed consent was waived by the same ethics committee of Henan Provincial People’s Hospital. All methods were conducted according to the relevant guidelines and regulations. Between January 2019 and May 2022, consecutive patients with intracranial aneurysms treated with the Tubridge flow diverter (stent) were identified by reviewing the medical records and were retrospectively enrolled. The inclusion criteria were patients with small and large ruptured or un-ruptured intracranial aneurysms confirmed by imaging examination treated with deployment of the Tubridge flow diverter at our hospital. The exclusion criteria were patients with infectious or traumatic intracranial aneurysms, history of surgical or endovascular intervention of intracranial aneurysms, and comorbidities of intracranial tumors or other diseases which affected endovascular management of intracranial aneurysms.

### Endovascular embolization

Dual antiplatelet therapy which comprised aspirin (100 mg/d) and clopidogrel (75 mg/d) was performed 3–5 days before embolization. Every patient underwent thromboelastography to detect the response of antiplatelet medication, and the dose of antiplatelet therapy was regulated in line with the test results. With the patient in the supine position under general anesthesia, access to the right femoral artery was conducted using the modified Seldinger technique. After successful sheath insertion into the femoral artery, systematic heparinization was started with 50 IU/kg unfractionated heparin through an intravenous bolus shot, followed by continuous infusion of heparin at a dose of 1000 IU per hour to maintain an activated clotting time (ACT) 2.5–3 times the baseline ACT during the embolization treatment. Cerebral angiography was carried out after a 6F guiding catheter was introduced through the arterial sheath to evaluate the aneurysm and parent artery. After measurement of the aneurysm and parent artery size, an appropriate Tubridge stent was selected and navigated to the aneurysm location under guidance of a micro-guide wire for deployment. The Tubridge stent was selected to ensure complete coverage of the aneurysm neck, with 3 mm beyond the distal or the proximal end of the aneurysm neck. For symptomatic (limb numbness and weakness, diplopia and blurred vision), giant, irregular, and ruptured aneurysms and those with a daughter aneurysm sac, coils were used to loosely embolize the aneurysm cavity to promote aneurysm thrombosis. When inserting the coils for loose packing, a micro-catheter was jailed between the Tubridge stent and the parent artery wall. After embolization, dual antiplatelet therapy with aspirin (100 mg/d) and clopidogrel (75 mg/d) was prescribed for three months before lifetme use of aspirin (100 mg/d) alone. Immediately after embolization, head computed tomography (CT) was conducted to evaluate possible subarachnoid hemorrhage.

### Follow-up

Follow-up was carried out with telephone contact, outpatient and inpatient visit or with cerebral angiography. Aneurysm occlusion status was evaluated using the O’Kelly-Marotta (OKM) scaling system^20^, and aneurysm filling grades were divided into grade A: complete (> 95%), B: incomplete (5%-95%), C: neck remnant (< 5%), and D: no filling (0%). The clinical prognosis was assessed with the modified Rankin scale (mRS) scores.

### Parameters for evaluation

The following parameters were assessed: patients’ age, sex, symptoms, past history of hypertension, diabetes mellitus, hyperlipidemia, smoking, alcohol abuse, rupture, aneurysm size, number and location, aneurysm neck size, diameter of parent artery, number of stents deployed, use of coils, procedural duration, periprocedural complications, follow-up duration, aneurysm occlusion status immediately after embolization and at follow-up, in-stent stenosis, and clinical outcomes. Wide aneurysm necks were defined as a neck ≥ 4 mm or the dome-neck-ratio ≤ 2. In-stent stenosis was defined as greater than 20% stenosis in the diameter of the stented segment on follow-up angiograms compared with the initial stented diameter on post-deployment angiograms immediately after stent deployment.

### Statistical analysis

The SPSS software (version 20.0, IBM, Chicago, IL, USA) was used for the statistical analysis. Continuous measurement data were expressed as mean ± standard deviation if in the normal distribution and tested with the paired t test or as median and interquartile range if not in the normal distribution and tested with the Mann Whitney *U* test. Categorical variables were presented as frequency and percentages and tested with the Chi square test. The significant *P* value was set at < 0.05.

## Results

### Subjects

Eighty-five patients were enrolled, including 35 (41.2%) males and 50 females (58.8%) with an age range of 17–77 (mean 56.7 ± 11.1) years (Table 1). Symptoms were dizziness in 29 (34.1% or 29/85) patients, headache in 28 (32.9% or 28/85), limb numbness and weakness in 8 (9.4% or 8/85), diplopia in 2 (2.4% or 2/85), and blurred vision in 2 (2.4% or 2/85). Sixteen (18.8% or 16/85) patients were incidentally found to have cerebral aneurysms. Hypertension was present in 53 (62.4% or 53/85) patients, diabetes mellitus in 9 (10.6% or 9/85), hyperlipidemia in 15 (17.6% or 15/85), smoking in 23 (27.1% or 23/85), and alcohol abuse in 19 (22.4% or 19/85).

**Table 1:** Baseline data of patients.

Variables	Data
Patients	
No	85
M/F	35/50
Age (y)	17–77 (56.7 ± 11.1)
With single aneurysms	65 (76.5%)
With multiple aneurysms	20 (23.5%)
Small aneurysms < 10 mm	50 (58.8%)
Large aneurysms > 10 mm	35 (41.2%)
Past history	
Hypertension	53(62.4%)
Diabetes mellitus	9(10.6%)
Hyperlipidemia	15(17.6%)
Smoking	23(27.1%)
Alcohol abuse	19(22.4%)
Aneurysm	
No	110
Size (mm)	2–30 (8.7 ± 6.1)
< 10 mm	65 (59.1%)
10–25 mm	43 (39.1%)
> 25 mm	2 (1.8%)
Neck size (mm)	2–10.6 (5.7 ± 2.3)
Single aneurysms	65 (59.1%)
Multiple aneurysms	45 (40.9%)
Aneurysm location	
ICA	61(55.5%)
PCOM	16(14.5%)
ACOM	1(0.9%)
MCA	1(0.9%)
Vertebral artery	24(21.8%)
Basilar artery	7(6.4%)
Aneurysm shape	
Dissecting	18 (16.4%)
Irregular	23 (20.9%)
Sacular	53 (48.2%)
Fusiform	7 (6.4%)
Tandem	2 (1.8%)
Giant	1 (0.9%)
With a daughter sac	6 (5.5%)

Data were presented in mean ± standard deviation or frequency and percentages.

*ICA* internal carotid artery, *PCOM* posterior communicating artery, *ACOM* anterior communicating artery, *MCA* middle cerebral artery.

### Aneurysms

There were totally 110 aneurysms, including one aneurysm in each of 65 (76.5% or 65/85) patients, two in 16 (18.8% or 16/85), 3 in 3 (3.5% or 3/85), and 4 in one (1.2% or 1/85). Five (5.9% or 5/85) patients initially presented with subarachnoid hemorrhage from aneurysm rupture, and aneurysms in the other 80 patients were unruptured. Sixty-one (55.5% or 61/110) aneurysms were located at the internal carotid artery (ICA), 16 (14.5% or 16/110) at the posterior communicating artery, one (0.9% or 1/110) at the anterior communicating artery, one (0.9% or 1/110) at the middle cerebral artery, 24 (21.8% or 24/110) at the vertebral artery, and 7 (6.4% 7/110) at the basilar artery (Table 1). The aneurysms ranged in size from 2 to 30 mm (mean 8.6, median 7, and interquartile range 6.7 mm), and the aneurysm neck was 2–10.6 (mean 5.7 ± 2.3) mm. Eighteen (16.4%) aneurysms were dissecting, 23 (20.9%) were irregular, 53 (48.2%) sacular, 7 (6.4%) fusiform, 2 (1.8%) tandem aneurysms, and 1 (0.9%) giant. Six aneurysms (5.5%) had a daughter aneurysm sac.

### Endovascular embolization

**Figure 1:**
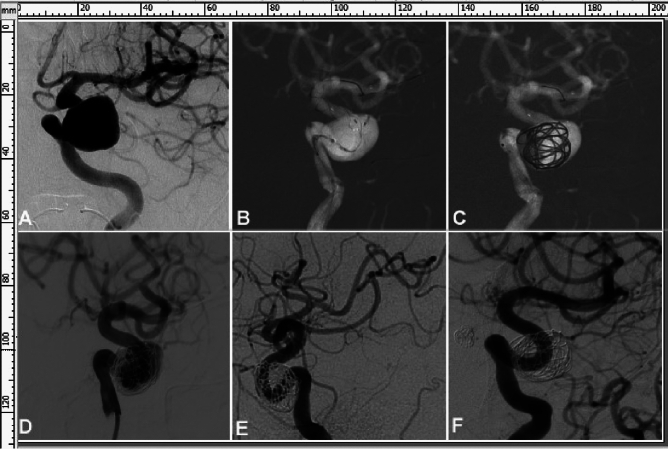
A patient in their early 60 s was diagnosed as having multiple cerebral aneurysms because of blurred vision, and one large aneurysm measuring 13.3 mm × 19.3 mm was found at the left internal carotid artery (ICA) cavernous segment and another measuring 6.5 mm × 6.2 mm was at the right ICA siphon segment. The left ICA cavernous aneurysm was treated with deployment of a Tubridge flow diverter of 5.5 mm × 35 mm followed by loose coil packing with 4 coils measuring 25 × 50, 25 × 50, 22 × 50, and 20 × 50, respectively. (**A**) The large aneurysm was shown at the left ICA cavernous segment. (**B**) A Tubridge flow diverter 5.5 mm × 35 mm was deployed with good apposition to the arterial wall. (**C**, **D**) Loose coil packing was performed. (**E**) Immediately after embolization, the large aneurysm was completely occluded. (**F**) At 17-month follow-up, the left ICA cavernous aneurysm remained completely occluded with patent parent artery.

**Figure 2:**
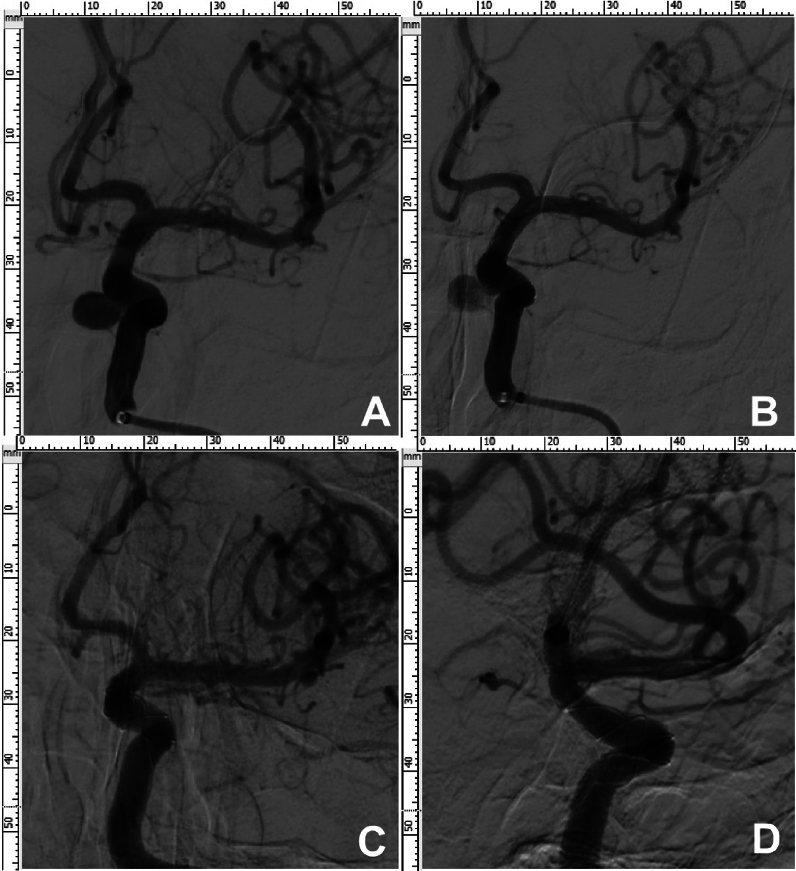
A patient in their early 50 s was found to have a left internal carotid artery (ICA) cavernous aneurysm because of dizziness. (**A**) A left ICA cavernous aneurysm measuring 7.2 × 6.7 mm was detected on digital subtraction angiography. (**B**) A Tubrdige flow diverter 5.5 mm × 35 mm was deployed to cover the aneurysm, and blood flow entering the aneurysm cavity was reduced at the end of treatment. (**C**, **D**) At 14-months follow-up angiography, the aneurysm was completely occluded, and the parent artery was patent without stenosis.

Endovascular embolization was successful in all patients (100%) with a total surgical time of 140.26 ± 52.37 (range 60–270) minutes (Figs. 1 and 2 and Table 2). Ninety-three Tubridge stents ranging from 2.5 × to 6.5 × 45 mm were deployed, including one stent in 77 (90.6% or 77/85) patients each and two stents in eight (9.4% or 8/85) patients each. Sixty (70.6% or 60/85) patents experienced stenting only while 25 (29.4% or 25/85) underwent stenting and adjunctive loose coil packing. One stent was deployed to cover two aneurysms in each of 12 patients, 3 aneurysms in one patient, and 4 aneurysms in one patient. In the remaining 79 (71.8% or 79/110) aneurysms, one stent was deployed to cover one aneurysm. Blood flow was significantly reduced from entering the aneurysm dome after stent deployment.

**Table 2:** Endovascular treatment and follow-up.

Variables	Data
Treatment	
Success rate	100%
No. of stents	93
Stenting only	60 (70.6%)
Adjunctive coiling	25 (29.4%)
One stent deployed	77 (90.6%)
Two stents deployed	8 (9.4%)
Periprocedural complications	
In-stent thrombosis	1 (1.2%)
Edematous conjunctiva	1 (1.2%)
Cerebral infarction	1(1.2%)
Immediate OKM grade	
A	10 (9.1%)
B	92 (83.6%)
C	8 (7.3%)
D	0
Follow-up	
Duration (m)	3–36 (15.3 ± 5.6)
No. of patients	67 (78.8%)
Symptoms	
Resolved	42 (60.9%)
Improved	22 (31.9%)
No change	5 (7.2%)
In-stent stenosis	
25%	3 (4.5%)
50%	2 (3.0%)
OKM occlusion grade	
B	2 (3.0%)
C	9 (13.4%)
D	56 (83.6%)

*OKM* O’Kelly-Marotta grading system.

Procedure-related complications occurred in three (3.5% or 3/85) patients, including in-stent thrombosis during embolization in one patient (1.2% or 1/85) which was treated with arterial injection of 8 ml Xinweining (tirofiban) followed by continuous pumping for 5 ml/h, conjunctiva edema on the right in one patient (1.2% or 1/85) caused by thrombosis to occlude relevant small arteries, and acute multiple cerebral infarctions confirmed by magnetic resonance imaging in one patient (1.2% or 1/85). After appropriate management, all three patients recovered without neurological sequela.

In addition, poor apposition of a Tubridge stent occurred in one (1/85 or 1.2%) patient with a 5 mm aneurysm at the posterior communicating artery segment. After deployment of a Tubridge 3.0 mm × 15 mm stent in this patient, the distal end of the stent did not appose well to the arterial wall even after repeated adjustment and massage with a microguide wire and a microcatheter. A Solitaire AB 4 mm × 20 mm stent was introduced to the distal end of the Tubridge stent and deployed to support the Tubridge stent. Immediate angiography showed good apposition of the stent, with patent stent and no stenosis. 

### Follow-up

Clinical follow-up was performed in all patients. The symptoms were resolved in 42 (60.9%, or 42/69) patients, improved in 22 (31.9%, or 22/69), not changed in 5 (7.2%, 5/69), and transiently worsened in 13 (18.8%, or 13/69). Angiographic follow-up was conducted in 67 (78.8%) patients 3–36 (mean 15.3 ± 5.6) months after the embolization. In 11 (16.4% or 11/67) patients, blood flow still entered the aneurysms with the OKM grade B in three (4.5%) patients and grade C in eight (11.9%), whereas complete occlusion (OKM grade D) was achieved in the other 56 (83.6% or 56/67) patients. Among 67 patients with angiographic follow-up, 20 patients had adjunctive coiling after deployment of the Tubridge stent. A greater proportion of patients (95% or 19/20) with adjunctive coiling achieved complete occlusion of the aneurysm (OKM grade D) compared with those (78.7% or 37/47) treated with Tubridge deployment alone (Table 3). In-stent stenosis was present in 5 (7.5%) patients, with approximately 25% stenosis in three (4.5%) patients and 50% in two (3.0%).

**Table 3:** Occlusion comparison of stent deployment alone with stenting + adjunctive coiling.

OKM grade	Stent alone	Stent + coiling	Total
Grade D	37 (78.7%)	19 (95%)	56 (83.6%)
Grade C	7 (14.9%)	1 (5%)	8 (11.9%)
Grade B	3 (6.4%)	0	3 (4.5%)
Total	47	20	67

*OKM* O’Kelly-Marotta grading system.

Subgroup analysis of patients with small and large aneurysms showed that the surgical time was significantly (*P* = 0.002) shorter in patients with small (< 10 mm in diameter) aneurysms than those with large (≥ 10 mm in diameter) ones (127.9 ± 47.2 vs. 163.7 ± 54.4 min). Significantly (*P* < 0.0001) more patients with large aneurysms experienced adjunctive coiling than those with small ones (62.2% vs. 12.3%) (Table 4). No significant (*P* > 0.05) difference existed in the periprocedural complications, number of patients with follow-up, in-stent stenosis, and OKM occlusion grade at follow-up between the two groups.

**Table 4:** Outcome comparison of patients with small and large aneurysms.

Variables	50 patients with 65 aneurysms < 10 mm	35 patients with 45 aneurysms ≥ 10 mm	*P*
Surgical time (min)	60–255 (127.9 ± 47.2)	60–270 (163.7 ± 54.4)	0.002
Coiling (n, %)	8 (12.3% or 8/65)	28 (62.2% or 28/45)	< 0.0001
Periprocedural complications (n, %)	2 (4%)	1 (2.9%)	> 0.05
Follow-up (n, %)	41(82%)	26 (74.2%)	> 0.05
In-stent stenosis (n)	3 (7.3%)	2 (7.7%)	> 0.05
OKM grade (n, %)			0.52
Grade D	37 (90.2%)	19 (73.1%)	
Grade C	3 (7.3%)	5 (19.2%)	
Grade B	1 (2.4%)	2 (7.7%)	

Data were presented in mean ± standard deviation or frequency and percentages.

*OKM* O’Kelly-Marotta grading system.

## Discussion

In this study investigating the safety and effect of Tubridge flow diverter deployment in the treatment of intracranial aneurysms, it was found that use of the Tubridge diverter could greatly decrease blood flow from entering the aneurysm, with a low periprocedural complication rate (3.5%), a high aneurysm occlusion degree and a low in-stent stenosis rate at follow-up. While large aneurysms may necessitate a longer surgical time and adjunctive coiling, no significant difference was detected in the periprocedural complications, in-stent stenosis and aneurysm occlusion degree between large and small aneurysms. This may indicate that the Tubridge flow diverter can be safely and effectively applied in the treatment of intracranial aneurysms, with good clinical and imaging occlusion outcomes.

Periprocedural complications in endovascular treatment of cerebral aneurysms may include in-stent thrombosis, arterial dissection, intra-procedural aneurysm rupture, and ischemic stroke. If few minor complications are encountered, it may mean that the device can be safely applied in the treatment of aneurysms. In a study investigating the effect of Tubridge stent in treating large and giant aneurysms^16^, the hemorrhagic stroke and aneurysm rupture rates were reported as high as 6.9% and 5.2%, respectively. In the IntrePED study with data from 793 patients among 17 centers, the intraparenchymal hemorrhage rate was reported as 2.4%, whereas the aneurysm rupture rate was 0.6%^21^. In a meta-analysis of 3125 patients^22^, the rates for calculated intraparenchymal hemorrhage and aneurysm rupture were, respectively, 2.9% and 1.8% for the total sample, 5.4% and 7.5% for giant aneurysms, and 2.1% and 1.3% for small and large aneurysms. In the IntrePED study^21^, similar intraparenchymal hemorrhage and aneurysm rupture rates were obtained, respectively, of 5.8% and 5.8% for giant internal carotid artery aneurysms, 2.6% and 0.5% for large aneurysms, and 1.9% and 0% for small aneurysms. In a study comparing the Pipeline (n = 39) and Tubridge (n = 53) flow diverters in treating intracranial aneurysms, the periprocedural complications have been reported to be 2.56% with one death (1/39) in the Pipeline and 3.77% of subarachnoid hemorrhage in the Tubridge flow diverter^1^. In a systematic review and meta-analysis of the Silk flow diverter for the treatment of intracranial aneurysms enrolling 14 studies^23^, the mortality rate, the overall thromboembolic complication rate, and the total hemorrhagic complication rate were reported, respectively, to be 2.84%, 6.06%, and 1.62%. In evaluating the safety and efficacy of the Surpass Evolve Flow diverter for intracranial aneurysms with 116 patients^24^, a procedural complication occurred in one case (0.9%) caused by wire-related perforation of an M3 branch distant from the giant aneurysm, and permanent neurological deficits were observed in 3 (2.5%) patients primarily due to early in-stent thrombosis, and no deaths were reported to link to the treatment. In a systematic review and meta-analysis of the Derivo Embolization Device for intracranial aneurysm treatment including five studies and 481 aneurysms^25^, the Derivo flow diverter was reported to have a periprocedural ischemic and hemorrhagic complication rate of 4.9%, a mortality rate 2.1%, and a delayed aneurysm rupture rate was reported 1/481 (0.2%). In assessing the flow-diverter devices in the treatment of intracranial aneurysms in a meta-analysis and systematic review enrolling twenty-nine studies with 1524 patients^26^, the overall technical failure and complication rate was 9.3%, and the rate of procedure-related complication was 14% and 6.6% for morbidity and mortality, respectively. Although our study had a complication rate of 3.5%, no major complications like death or subarachnoid hemorrhage had occurred. Fewer complications in this study were probably due to accumulation of experience gained in applying the Tubridge device in the treatment of dozens of patients with intracranial aneurysms prior to this study.

For aneurysm occlusion, good OKM grades (grade C and D) indicate good occlusion and treatment effect. For the FRED flow diverter, the complete aneurysm occlusion (grade D) rate has been reported to range from 66 to 87% or even 100%, with the complete occlusion rate being continuously increased with extension of the follow-up time^27–29^. In reporting angiographic follow-up outcomes of 71 patients with 77 large or giant cerebral aneurysms treated with the Pipeline flow diverter (excluding 7 patients with parent artery occlusion)^30^, complete aneurysm occlusion (OKM grade D) was achieved in 64.9% aneurysms 6 months after embolization, 76.6% after 1 year, and 77.9% at 3-years follow-up, with the trend of occlusion being gradually increased over time. Nonetheless, the complete aneurysm occlusion rate at different follow-up periods had also been reported to range from 81.9 to 93.4% for the Pipeline stent in the treatment of intracranial aneurysms^31–33^. In a study comparing the effect of the Pipeline and Tubridge stents for the treatment of cerebral aneurysms^1^, the complete aneurysm occlusion rate was 77.42% for the Pipeline stent at a mean follow-up time of 9.7 months with no in-stent stenosis and 85.71% for the Tubridge stent at a mean follow-up time of 9.1 months with in-stent stenosis in three patients (3/42). Different complete occlusion rates may be associated with different follow-up durations and ages of the patients. With increase of the follow-up time, more aneurysms will obtain complete occlusion, which involves intra-aneurysm thrombosis and aneurysm neck endothelialization^34^. In our study, aneurysms treated with adjunctive coiling achieved a higher complete occlusion rate compared to those without coiling (95% vs. 78.7%), and smaller aneurysms obtained a higher complete occlusion rate compared to those larger ones (90.2% vs. 73.1%) at a mean follow-up of 15.3 months.

In one study investigating the effect of the Pipeline stent for the treatment of intracranial aneurysms^33^, the success rate was reported to be 99.3%, and the mean total surgical time was 78.4 ± 40.3 min (range 20–217), with additional coiling used only in 5 (3.5%) cases. In our study, the total surgical time was 140.26 ± 52.37 (range 60–270) minutes with a significantly shorter surgical time for patients with smaller aneurysms than those with larger ones. Significantly more patients with larger aneurysms experienced adjunctive coiling than those with smaller ones (62.2% vs. 12.3%). Coiling was usually performed in ruptured, symptomatic, large and giant aneurysms or those with a daughter sac so as to promote thrombosis within the aneurysm cavity. More aneurysms treated with stenting and adjunctive coiling achieved complete occlusion (95%) compared to those with stenting only (78.7%). No significant (*P* > 0.05) difference existed in the periprocedural complications, numbers of patients with follow-up, in-stent stenosis, and OKM aneurysm occlusion grade at follow-up between patients with small and large aneurysms.

The p64 flow diverter has been extensively investigated in clinical application for treating cerebral aneurysms^35–37^. In one study exploring progressive volume decrease and long-term aneurysmal collapse after deployment of the p64 flow diverter for 36 giant and symptomatic intracranial aneurysms^37^, significant aneurysmal shrinkage was observed in 32 aneurysms at 24-months follow-up compared with 25 aneurysms at 6-month follow-up. After treatment, all 13 patients with the third cranial nerve palsy demonstrated improvements, and the total post-treatment complication rate was 8.3% with no deaths. In a study investigating the mid- to long-term outcomes of the p64 flow diverter in the treatment of 72 patients with 72 cerebral aneurysms^36^, complete aneurysm occlusion degrees were also shown to be time-dependent. At six-month follow-up, the complete aneurysm occlusion rate with the OKM grade D was achieved in 76.3% (n = 55 aneurysms), which was increased to 91.4% (n = 64 aneurysms) at 12 months and 94.4% (n = 67) at 24 months after deployment of the p64 flow diverter. Permanent disability caused by delayed aneurysm rupture took place in one patient (1.38%), with no mortality being reported. In an international, prospective, multicenter, single-arm post-market study exploring the safety and effectiveness of the p64 flow modulation device in the treatment of 420 patients with intracranial aneurysms measuring 6.99 ± 5.28 mm in the dome width^35^, adjunctive coiling was performed in 14.0% of the patients, a composite morbidity/mortality rate was 2.42% at a follow-up duration of 145 ± 43 days, and complete aneurysm occlusion was achieved in 83.7% of the patients at a follow-up of 375 ± 73 days. This multicenter study has demonstrated favorable outcomes of the p64 flow diverter. Our study had comparable outcomes even though the periprocedural complication rate was slightly higher than that in the multicenter study^35^.

Some limitations existed in this study, including the retrospective and single-center study design, a relatively small cohort of patients, Chinese patients enrolled only, no control, no randomization, and a limited follow-up period, which may all have a bad impact on the generalization of the outcome. The period of this study was between January 2019 and May 2022, and patients receiving the Tubridge stenting during this short period were screened and enrolled, which accounted for the limited number of patients and limited follow-up duration. Over time, more patients would have a longer follow-up period for better outcome analysis. Future prospective, randomized, controlled studies involving multiple medical centers with a large cohort of patients of different races and ethnicities are necessary to obtain better outcomes.

In conclusion, the Tubridge diverter can be safely and efficiently applied for the treatment of small and large intracranial aneurysms, with a low periprocedural complication rate, a high aneurysm occlusion degree, and a low in-stent stenosis rate at follow-up even though large aneurysms may necessitate a longer surgical time and adjunctive coiling.

## Data Availability

The datasets used and/or analysed during the current study are available from the corresponding author on reasonable request.
